# Characterization of DNA topoisomerase I in three SN-38 resistant human colon cancer cell lines reveals a new pair of resistance-associated mutations

**DOI:** 10.1186/s13046-016-0335-x

**Published:** 2016-03-31

**Authors:** Niels Frank Jensen, Keli Agama, Amit Roy, David Hersi Smith, Thomas D. Pfister, Maria Unni Rømer, Hong-Liang Zhang, James H. Doroshow, Birgitta R. Knudsen, Jan Stenvang, Nils Brünner, Yves Pommier

**Affiliations:** Department of Veterinary Disease Biology, Faculty of Health and Medical Sciences, Section for Molecular Disease Biology, University of Copenhagen, Strandboulevarden 49, DK-2100 Copenhagen, Denmark; National Institutes of Health, National Cancer Institute, Center for Cancer Research, Laboratory of Molecular Pharmacology, 37 Convent Drive, Building 37, Room 5068, Bethesda, MD 20892-4255 USA; Department of Molecular Biology and Genetics, Aarhus University, C.F. Møllers Allé 3, Building 1130, DK-8000 Aarhus C, Denmark; Department of Biotechnology, National Institute of Pharmaceutical Education and Research (NIPER), Hajipur, Vaishali 844102 India; Dako Denmark A/S, R&D, Produktionsvej 42, DK-2600 Glostrup, Denmark; Laboratory of Human Toxicology and Pharmacology, Applied/Developmental Directorate, Leidos Biomedical Research, Inc., Frederick National Laboratory for Cancer Research, Frederick, MD 21702 USA; Department for Clinical Physiology and Nuclear Medicine, Frederiksberg Hospital, Nordre Fasanvej 57, DK-2000 Frederiksberg C, Denmark

**Keywords:** DNA topoisomerase I, *TOP1*, SN-38, Irinotecan, Resistance, Colon cancer, Mutation

## Abstract

**Background:**

DNA topoisomerase I (Top1) is a DNA unwinding protein and the specific target of the camptothecin class of chemotherapeutic drugs. One of these, irinotecan, acting through its active metabolite SN-38, is used in the treatment of metastatic colorectal cancer. However, resistance to irinotecan represents a major clinical problem. Since molecular alterations in Top1 may result in resistance to irinotecan, we characterized Top1 in three human colon cancer cell lines with acquired resistance to SN-38.

**Methods:**

Three SN-38 resistant (20–67 fold increased resistance) cell lines were generated and compared to wild-type parental cells with regards to: *TOP1* gene copy number and gene sequence, Top1 expression (mRNA and protein), Top1 enzymatic activity in the absence and presence of drug, and Top1-DNA cleavage complexes in drug treated cells. *TOP1* mutations were validated by PCR using mutant specific primers. Furthermore, cross-resistance to two indenoisoquinoline Top1-targeting drugs (NSC 725776 and NSC 743400) and two Top2-targeting drugs (epirubicin and etoposide) was investigated.

**Results:**

Two of three SN-38 resistant cell lines carried *TOP1* gene copy number aberrations: A *TOP1* gene copy gain and a loss of chromosome 20, respectively. One resistant cell line harbored a pair of yet unreported *TOP1* mutations (R364K and G717R) in close proximity to the drug binding site. Mutant *TOP1* was expressed at a markedly higher level than wild-type *TOP1*. None or very small reductions were observed in Top1 expression or Top1 activity in the absence of drug. In all three SN-38 resistant cell lines Top1 activity was maintained in the presence of high concentrations of SN-38. None or only partial cross-resistance were observed for etoposide and epirubicin, respectively. SN-38 resistant cells with wild-type *TOP1* remained sensitive to NSC 743400, while cells with mutant *TOP1* was fully cross-resistant to both indenoisoquinolines. Top1-DNA cleavage complex formation following drug treatment supported the other findings.

**Conclusions:**

This study adds to the growing knowledge about resistance mechanisms for Top1-targeting chemotherapeutic drugs. Importantly, two yet unreported *TOP1* mutations were identified, and it was underlined that cross-resistance to the new indenoisoquinoline drugs depends on the specific underlying molecular mechanism of resistance to SN-38.

## Background

In metastatic colorectal cancer (mCRC), current chemotherapeutic treatment options consist of either 5-fluorouracil and folinic acid with oxaliplatin (FOLFOX) or 5-fluorouracil and folinic acid with irinotecan (FOLFIRI) [1, 2]. However, the response rate to these regimens is only in the range of about 30–55 %. Resistance to chemotherapy and drug-induced side effects are major limitations [3, 4], and the 5-year survival rate is less than 10 % [5]. One way to improve therapeutic efficacy is to introduce biomarkers to identify patients with a high likelihood of benefiting from drug treatment (see e.g. [6, 7]).

Irinotecan belongs to the camptothecin class of chemotherapeutic drugs and is a pro-drug of the active metabolite SN-38. Camptothecins selectively target DNA topoisomerase I (Top1)-DNA cleavage complexes which form in the vicinity of replication and transcription complexes to unwind DNA [8–10]. Top1 is a 765 amino acid residue protein encoded by the 21-exon gene, *TOP1* located on the long arm (q) of chromosome 20. Top1 binds supercoiled DNA, nicks a DNA strand allowing its rotation around the intact strand, and then religates the DNA [8, 11]. Camptothecins bind and stabilize the Top1-DNA cleavage complexes, thus leading to DNA damage when replication or transcription occurs [8]. Similarly, DNA topoisomerase II (Top2) is the target of other classes of chemotherapeutic drugs, including the anthracylines and etoposide [8, 12, 13].

As Top1 is the direct target of SN-38, the active metabolite of irinotecan, it has been extensively studied as a possible mediator of resistance or as a predictive marker in mCRC. Top1 can be examined in several different ways; gene copy number aberrations and genetic mutations, mRNA and protein expression levels, and enzyme activity levels (see e.g. [6]). Studies have been performed both at the pre-clinical cellular level (e.g. [14–17]) and using clinical tumor samples (e.g. [17–20]). Positive correlation between Top1 protein level and gene copy number or mRNA level has been observed in several studies [14, 21, 22]. In cell-based studies, high Top1 expression and enzyme activity have generally been associated with sensitivity to camptothecins, whereas low Top1 is a common resistance mechanism [15, 16, 23–26]. In addition, mutations or DNA methylation of the *TOP1* gene have been associated with resistance to camptothecins [27, 28]. Most mutations have been identified in cultured cells [27], and rarely in clinical patient material [29]. The largest clinical study investigating Top1 as a predictive marker of irinotecan treatment in mCRC to date is the UK FOCUS trial [18, 30]. High tumor Top1 protein expression was found to correlate significantly with therapeutic benefit from irinotecan. However, a similar study, the Dutch CAIRO trial [31, 32], was not able to replicate this finding.

In recent years, new classes of non-camptothecin Top1-targeting drugs have reached clinical development, e.g. the indenoisoquinolines, the dibenzonaphtyridinones and the indolocarbazoles [8, 33, 34]. Compared to camptothecins, indenoisoquinoline drugs are chemically stable, bind Top1-DNA cleavage complexes at other DNA sequences, form less reversible drug-Top1-DNA cleavage complexes and are not substrates of common multi-drug resistance efflux pumps [34, 35].

In the present study we undertook a thorough investigation of the Top1 status in three human colon cancer cell lines with acquired resistance to SN-38 developed through approximately 9 months of drug exposure [36]. We investigated the Top1 gene copy number, genetic sequence, mRNA expression level, protein expression level, enzyme activity and formation of Top1-DNA cleavage complexes following drug treatment. In addition we tested the cross-resistance to two non-camptothecin Top1-targeting drugs as well as two drugs targeting Top2.

## Methods

### Cell culture

The cell lines HCT116 and HT29 were obtained from the NCI/Development Therapeutics Program, while LoVo was obtained from the American Tissue Culture Collection. Cells were maintained at 37 °C, 5 % CO_2_ in RPMI 1640 + Glutamax growth medium (Invitrogen, Nærum, Denmark) supplemented with 10 % fetal calf serum (Invitrogen). SN-38 resistant cell lines were generated in our laboratory by exposing three colon cancer cell lines to gradually increasing drug concentrations for 8–10 months [36]. The cells were maintained in drug-free growth medium for at least 1 week and at most 4 weeks prior to any experiments.

### Chemotherapeutic drugs

SN-38 (Sigma-Aldrich, Copenhagen, Denmark) was purchased and dissolved in dimethyl sulfoxide (DMSO) at a concentration of 10 mM and stored at -20 °C. The indenoisoquinoline drugs NSC 725776 (LMP776) and NSC 743400 (LMP400), provided by the laboratory of Dr. Yves Pommier, were dissolved in DMSO at a concentration of 5 mM and stored at -20 °C. Epirubicin (2 mg/ml, Actavis Nordic A/S, Gentofte, Denmark) and etoposide (20 mg/ml, Pfizer, New York, USA) were purchased and stored at -20 °C. Drugs were diluted in growth medium immediately prior to use.

### Drug sensitivity MTT assay

In vitro drug sensitivity was determined using the MTT (methylthiazolyldiphenyl-tetrazolium bromide) assay. Cells were seeded in 96-well plates, and a range of drug concentrations was added the following day. Following 48 h of drug exposure, the medium was discarded and the plates were incubated with medium containing MTT (0.5 mg/ml, Sigma-Aldrich) for 3 h. Acidified (0.02 M HCl) sodium dodecyl sulphate (20 %, Sigma-Aldrich) was added to dissolve the formed formazan. Optical density at 570 nm (and 670 nm for background) was measured, and the cell viability was calculated in percent compared to untreated cells. Experiments were repeated three times and the mean IC50-value ± standard deviation was determined. Relative resistance for each resistant cell line was calculated by dividing the mean IC50-value of the resistant cell line by the mean IC50-value of the corresponding parental cell line.

### RNA purification and mRNA analysis

The RNA purification and *TOP1* mRNA analysis is previously described [36]. Briefly, RNA was harvested from each cell line in triplicate using TRIzol Reagent (Invitrogen) and quantified using a Nano-Drop ND-1000 (Thermo Scientific, Waltham, USA). The Top1 mRNA level was obtained from a gene expression microarray analysis (Human Gene Expression Microarrays G4112F, Agilent Technologies, Santa Clara, USA) done in triplicate calculating mean ± standard deviation.

### Metaphase preparation

Metaphase preparation has previously been described [37]. Briefly, upon reaching a confluence of approximately 70 %, colcemid (Invitrogen) was added to cell cultures. After 2 h at 37 °C, cells were harvested and a hypotonic treatment was performed (0.075 M KCl) for 10 min. Cells were fixed (fixative: 3:1 vol/vol absolute methanol and glacial acetic acid) and the suspension was dripped onto glass slides.

### Fluorescence-in-situ-hybridization (FISH) gene copy number analysis

The *TOP1*/Centromere-20 (CEN-20) probe combination and relevant protocol has previously been described [17]. FISH reagents were from the Cytology FISH Accessory Kit (K5499) and the Histology FISH Accessory Kit (K5799) (Dako A/S, Glostrup, Denmark). Metaphase specimens were fixed in 3.7 % formaldehyde, washed, dehydrated and air dried. Once dry, FISH probe was loaded onto slide, denatured and hybridized overnight. Excess probe was removed by washing in stringency buffer. Slides were washed, dehydrated, air dried and mounted. To determine the presence and mechanism of *TOP1* copy number alteration in cell lines, signal locations and numbers were noted for 50 metaphases for each cell line at 1000x magnification.

### TOP1 DNA sequencing

Six primer sets covering the full coding region of human *TOP1* (NCBI Reference Sequence: NM_003286.2) were obtained: (1) 5′-CTCAGCCGTTTCTGGAGTCT-3′ (forward) and 5′-TCAGCATCATCCTCATCTCG-3′ (reverse) (593 bp); (2) 5′-CGAAAAGAGGAAAAGGTTC-3′ and 5′-GGGCTCAGCTTCATGACTTT-3′ (488 bp); (3) 5′-CCACCATATGAGCCTCTTCC-3′ and 5′-CCTTGTTATCATGCCGGACT-3′ (544 bp); (4) 5′-AGAGCCTCCTGGACTTTTCC-3′ and 5′-GACCATCCAACTCTGGGTGT-3′ (497 bp); (5) 5′- TTCGTGTGGAGCACATCAAT-3′ and 5′-GACCTTGGCATCAGCCTTAG-3′ (503 bp); (6) 5′-CGAGCTGTTGCAATTCTTTG-3′ and 5′-ACCACACTGTTCCTCTTCAC-3′ (472 bp). The primer sequences were obtained from [38] and primers purchased from Eurofins MWG Operon (Ebersberg, Germany). Total RNA was purified from cells as described above and converted to cDNA (SuperScript VILO cDNA Synthesis Kit, Invitrogen) according to the manufacturer’s instructions, using 100 ng RNA for each reaction. PCR products were amplified (HotStarTaq Master Mix Kit, Qiagen, Venlo, Netherlands) using the six primer sets described above. PCR products were assessed on a 1.5 % agarose gel (electrophoresis: 120 V, 2 h) and diluted to approximately 10 ng/μl. PCR products were shipped to and sequenced in both directions using the above mentioned forward and reverse primers by a company (Prepaid Plate Kit PCR Products, Eurofins MWG Operon). PCR products containing identified mutations were re-sequenced once. Sequences were compared between cell lines using multiple sequence alignment (Clustal Omega tool, EMBL-EBI, www.ebi.ac.uk).

### PCR validation of TOP1 mutations

Primers with similar melting temperatures specific for wild-type (wt) or mutant (mt) sequences were designed: 5′-GGACTTTTCCGTGGCC-3′ (364wt, forward); 5′-TGGACTTTTCCGTGGCT-3′ (364mt, forward); 5′-GATAATTGAGTTTGGAGGTTCC-3′ (717wt, reverse); 5′-CAGATAATTGAGTTTGGAGGTTCT-3′ (717mt, reverse). 364 and 717 refers to the amino acid residue positions of the mutations. The mutated nucleotides are underlined in the primer sequences. The primers were purchased from Eurofins MWG Operon. cDNA from cell lines were prepared as described above, and PCR products were amplified as described above using combinations of the wild-type and mutation specific primers. PCR products (10 μl per sample) were loaded on a 2 % agarose gel along with a DNA ladder (MassRuler DNA Ladder, Low Range, Fermentas, Thermo Scientific) and negative controls (PCR reactions run on RNAse-free water). Gel electrophoresis was done as described above and the gel was photographed under UV illumination.

### Protein purification and Western blotting

Cells were trypsinized, resuspended and washed in cold PBS and pellet frozen at -80 °C. Cell pellets were resuspended in lysis buffer [1 % SDS, 10 mmol/L Tris (pH 7.4), 40 μL of 25x protease inhibitors (Roche, Basel, Switzerland), 10 μL phosphatase inhibitors (Sigma), 1 mL water]. Samples were sonicated (15 s), kept for 5 min on ice and heated for 5 min at 95 °C. Total protein was determined using the Lowry method, i.e. absorbance measurement and using a standard curve of bovine serum albumin in sample buffer. Samples were diluted and 10 μg of total protein per sample were applied on a 4–20 % gradient gel (Novex, Invitrogen) together with a marker (SeaBlue Plus2 Pre-Stained Standard, Invitrogen), and subjected to electrophoresis (120 V, 1.5–2 h). Gel was transferred to a polyvinylidene difluoride (PVDF) membrane by semi-dry blotting 5 V overnight. Membrane was blocked for 1 h at room temperature in 5 % milk in PBS-Tween20 buffer. Subsequently, the membrane was stained with primary antibody, either anti-Top1 (C21, 1:1000, BD Biosciences Pharmingen, Franklin Lakes, NJ, USA) or anti-actin (1:5000, ab3280, Abcam, Cambridge, UK) (loading control), by incubation overnight at 4 °C. Membrane was washed three times, and incubated with HRP-conjugated secondary antibody (1:10000, sheep anti-mouse, GE Healthcare, Little Chalfont, UK) for 1 h at 30 °C. Subsequently, the membrane was washed three times and substrate (SuperSignal West Pico Chemiluminescent Substrate, Pierce, Thermo Scientific) was added according to the manufacturer’s instructions. Signal was developed in the dark room using a photographic film. Western blots were replicated.

### Top1 ELISA assay

Cells were trypsinized, resuspended and washed in cold PBS and pellet frozen at -80 °C. The Top1 ELISA was performed as previously described [39]. Cell pellets were sonicated in lysis buffer and protein concentration was determined by BCA assay. Mouse anti-Top1 monoclonal antibody clone C21.1 (BD Biosciences Pharmingen, 1:1000), was used as the capture antibody. Pure rTop1 (EMD Biosciences, Inc.) was used as to make the standards. Samples and standards were diluted in PBS-casein and incubated overnight at 2 °C to 8 °C. Rabbit anti-Top1 polyclonal antibody Ab28432 (Abcam, 1:500 in PBS-casein) was used as the probe followed by the addition of extra serum-absorbed goat-anti-rabbit horseradish peroxidase conjugate (KPL, 1:1000). Probe antibody and HRP-conjugate were pre-incubated with mouse serum (Sigma Aldrich, 1:1000) to lower background signal. Finally, Pico-ELISA substrate (Thermo Scientific Pierce) was added and chemiluminescence was measured on an Infinite 200 M (Tecan Group Ltd.). Top1 levels were normalized to 1 μg protein load.

### Top1 enzyme activity assay

Cells were trypsinized, resuspended and counted, and for each cell line 1 million cells were pipetted to each of three eppendorf tubes on ice. Cells were pelleted (5 min centrifugation, 300 g, 4 °C) and snap-frozen in dry ice and ethanol and stored at -80 °C until analysis. Nuclear extracts were prepared essentially as previously described [40], and Top1 activity measured in titration experiments with or without added SN-38 (at concentrations stated in the text) using the standard Rolling circle Enhanced Enzyme Activity Detection (REEAD) protocol as previously described [40, 41]. The activity was calculated in terms of numbers of Top1 specific signals relative to the amount of signals resulting from the addition a known concentration of control circles, as previously described [40].

### Drug treatment and detection of Top1-DNA cleavage complexes using alkaline elution

Top1-DNA cleavage complexes (DPCs) were detected using alkaline elution as previously described [42]. In brief, cells seeded in flasks were radiolabeled overnight with 0.02 μCi/ml [^14^C]thymidine and chased with radioisotope-free medium 4 h before drug treatment. Cells were treated with either SN-38 (10, 1 or 0.1 μM) or NSC 743400 (1 μM) for 1 h. Untreated cells were included as controls. Cells were harvested by scraping and quickly pipetted to tubes on ice. Cell aliquots were placed in ice-cold PBS and irradiated with 3000 rad to break the DNA. Cells were layered onto polyvinylchloride-acrylic copolymer (protein adsorbing) filters and lysed with LS-10 (2 M NaCl, 0.2 % sarkosyl, and 0.04 M disodium EDTA, pH 10). DNA was eluted from the filters with tetrapropylammonium hydroxide-EDTA (pH 12.15). After elution, filters were incubated for 1 h at 65 °C with 1 M HCl and an additional hour at room temperature in the presence of 0.4 M NaOH. Radioactivity in fractions and filters was measured with a liquid scintillation analyzer (2200A Tri Carb Scintillation Analyzer, Packard Instruments, Meridien, USA) and the fraction of DNA retained on the filter at each time point was calculated. The results were converted to rad-equivalent (a measure of DNA-protein crosslinks). Experiments were done twice each with two technical replicates.

### Statistical analyses

mRNA expression values in triplicate was compared between corresponding parental and resistant cell lines using unpaired Student’s t-tests. Formation of DNA crosslinks in different cell lines following drug treatment (either SN-38 or NSC 743400; 1 μM) was compared using paired Student’s t-tests. A *p*-value less than 0.05 was considered significant.

### Bioinformatics analysis of identified TOP1 mutations

The evolutionary conservation of selected amino acids in the Top1 protein was analyzed across seven different species (human, rhesus macaque, mouse, cow, frog, zebrafish and arabidopsis) using multiple sequence alignment (Clustal Omega tool, EMBL-EBI, www.ebi.ac.uk).

## Results

### Colon cancer cell lines resistant to SN-38

SN-38 resistant human colon cancer cell lines were generated from the three human colon cancer cell lines HCT116, HT29 and LoVo by approximately 9–10 months exposure to increasing concentrations of SN-38 [36]. Resistance to SN-38 in these cell lines were 67-, 55- and 20-fold, respectively, when comparing IC50-values for HCT116-SN38, HT29-SN38 and LoVo-SN38 to IC50-values of their the corresponding parental cell lines HCT116-Wt, HT29-Wt and LoVo-Wt [36].

### TOP1 gene copy number aberrations in SN-38 resistant cells identified by FISH

**Fig. 1:**
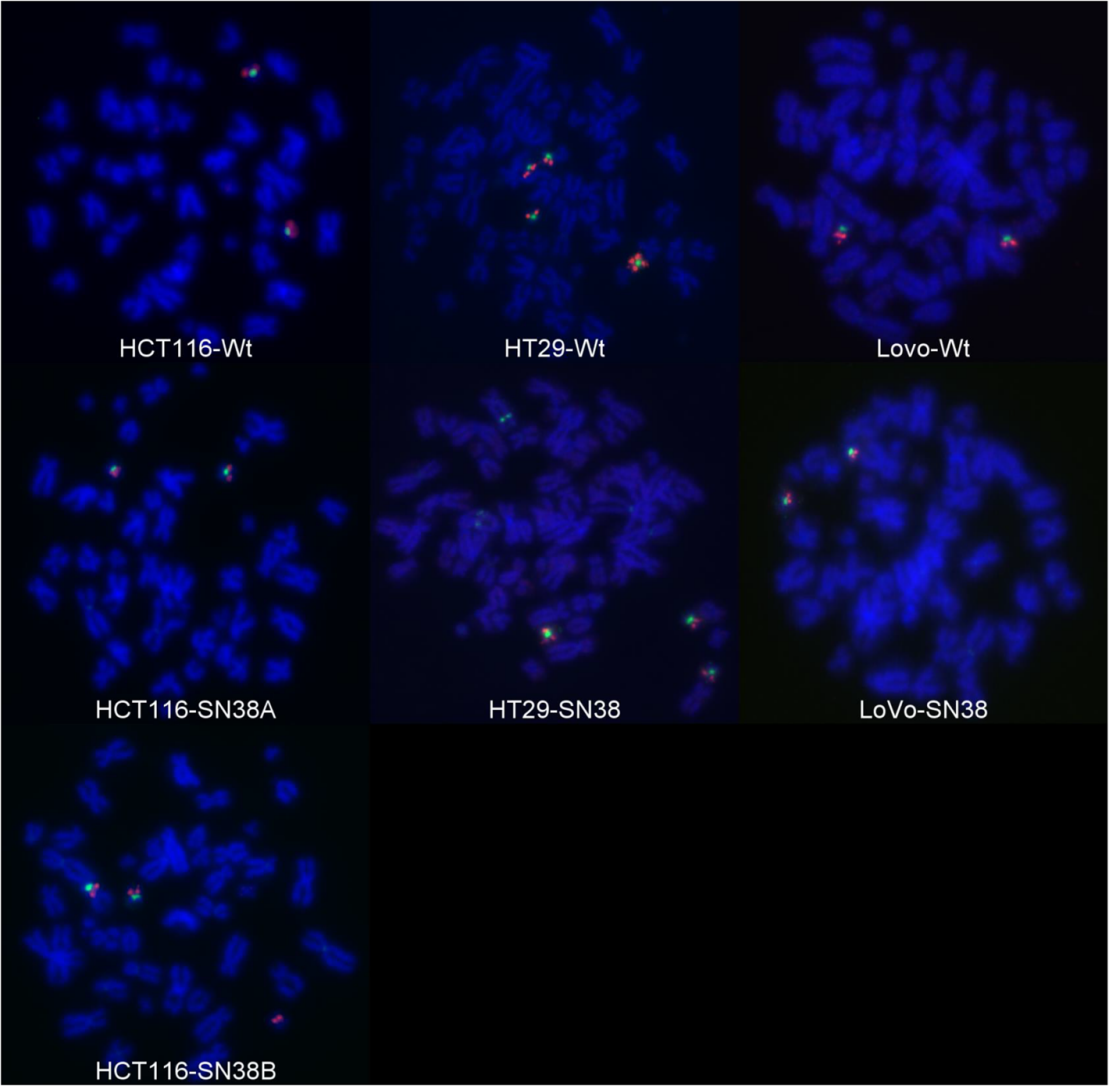
FISH analysis of *TOP1* (*red*) and CEN-20 (*green*) aberrations in parental and SN-38-resistant cells. Shown are representative cell metaphase FISH images of parental (HCT116-Wt, HT29-Wt and LoVo-Wt) and SN-38 resistant (HCT116-SN38, HT29-SN38 and LoVo-SN38) cell lines. HCT116-SN38A and –B designate the two subpopulations observed in the HCT116-SN38 cell line. See text and Table 1 for details. Note: Due to the existence of two chromatids in each metaphase chromosome, the observed number of gene signals is double that of what is observed in an interphase nucleus

*TOP1* gene copy number was determined using a *TOP1*/CEN-20 FISH probe combination, where CEN-20 is used as a reference (*TOP1* is located at 20q12-q13.1), in both the parental and resistant cells (see Table 1 and Fig. 1). LoVo-Wt and HCT116-Wt, both had two normal copies of chromosome 20, generating two *TOP1* and CEN-20 signals. In HCT116-SN38, 46 % of scored metaphases harbored two normal copies of chromosome 20, whereas in the remaining 54 %, an additional *TOP1* signal was observed on a chromosome that did not contain a CEN-20 signal. No aberrations were detected in the LoVo-SN38 cell line. In HT29-Wt, three normal copies of chromosome 20 were detected, along with four *TOP1* signals surrounding CEN-20, indicative of 20q isochromosome formation. A similar pattern was observed in HT29-SN38; however only two copies of chromosome 20 were detected, indicating a chromosome 20 loss. Additionally, a CEN-20 signal was detected in a chromosome without *TOP1* signal. It should be noted that this signal was observed outside of the centromere on this unknown chromosome (see Fig. 1).

**Table 1:** *TOP1*/CEN-20 FISH analysis

Cell line	*TOP1*	CEN-20	*TOP1*/CEN-20 ratio	Description of aberration^a^
HCT116-Wt	2	2	1	None
HCT116-SN38	A: 2, B: 3	A: 2, B: 2	A: 1, B: 1.5	Two subpopulations; A (46 %): No aberration, B (54 %): *TOP1* gain
HT29-Wt	5	4	1.25	20q isochromosome formation
HT29-SN38	4	4	1	20q isochromosome formation, loss of chromosome 20 and gain of CEN-20 on chromosome without *TOP1*
LoVo-Wt	2	2	1	None
LoVo-SN38	2	2	1	None

Wt (wild-type) designates the parental cell lines

^a^For details see text

### TOP1 sequencing reveals a yet unreported pair of highly expressed mutations in SN-38 resistant cells

**Fig. 2:**
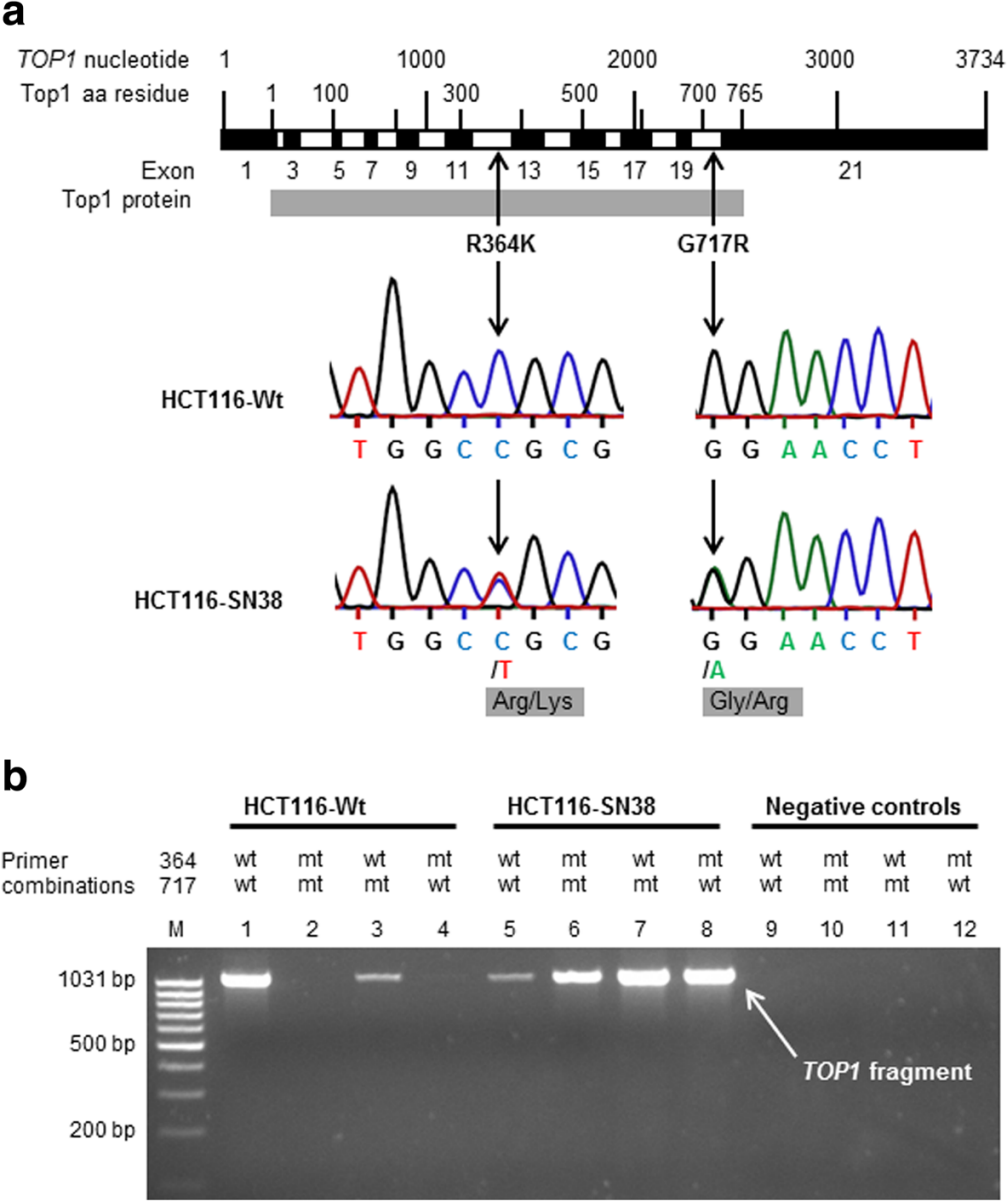
TOP1 mutations in HCT116 SN-38 resistant cells. **a** Location and sequencing chromatograms for the detected *TOP1* mutations, R364K and G717R. Both are heterozygous and detected in the SN-38 resistant HCT116-SN38 cell line. Top1 is a 765 amino acid (aa) residue protein [11, 46]. **b** Validation of mutations using PCR and combinations of wild-type (wt) and mutant (mt) specific primers at the 364 and 717 amino acid residue positions of *TOP1*. Shown is gel electrophoresis of the amplified PCR products. M is the DNA ladder; 1–4 are PCR products made from total cDNA from the parental HCT116-Wt cell line, and 5–8 are from the SN-38 resistant HCT116-SN38 sub-line. Negative controls (9–12) are the specified primer combinations. The band representing the amplified fragment of *TOP1* cDNA should have a size of 1059 bp [(717-364) x 3 bp per amino acid residue]

The DNA sequence of the full coding region of *TOP1* in parental and SN-38 resistant cells were obtained by PCR amplification using six primer sets followed by bidirectional sequencing of the PCR products. Sequences of corresponding parental and resistant cells were compared to identify acquired mutations in *TOP1* (see Table 2). No mutations were detected in HT29-SN38 or LoVo-SN38. In HCT116-SN38 two non-synonymous, heterozygous mutations were detected, namely c.1336C > T (corresponding to R364K, arginine to lysine change) and c.2395G > A (corresponding to G717R, glycine to arginine change) (see Fig. 2a for sequencing chromatograms). These mutations were confirmed by re-sequencing. Multiple sequence alignment demonstrated the mutations to correspond to evolutionarily conserved residues (data not shown). R364 was conserved in seven of seven analyzed species, while G717 was conserved in six of seven species (all except zebrafish; S717).

**Table 2:** Mutations in *TOP1*

Cell line	Mutations detected^a^	Location^b^
HCT116-SN38	c.1336C > T (R364K), c.2395G > A (G717R)	Exon 12, exon 20
HT29-SN38	None	-
LoVo-SN38	None	-

Both mutations are heterozygous

^a^Comparing sequences of resistant and parental cell lines

^b^Information from the NCBI (www.ncbi.nlm.nih.gov)

The identified *TOP1* mutations were validated using PCR primers designed to be specific for wild-type (wt) or mutant (mt) sequences at amino acid residue positions 364 and 717 in *TOP1*. A fragment of *TOP1* was amplified by PCR using combinations of primers and cDNA (made from mRNA) from parental or resistant cells (see Fig. 2b). Parental HCT116-Wt cells highly expressed wild-type *TOP1* (using wild-type primers at both positions 364 and 717), while no fragment was amplified using the combination of mutant primers. A relatively weak band was seen using 364wt-717mt primers, which might represent unspecific primer binding or a weak expression of mutant G717R *TOP1* in parental cells. On the other hand, SN-38-resistant HCT116-SN38 cells only weakly expressed wild-type *TOP1*, while 364mt-717mt and the wt-mt combinations of *TOP1* were highly expressed. Single-mutant (wt-mt or mt-wt combinations of primers) *TOP1* appeared to be more highly expressed than double-mutant (mt-mt) *TOP1*.

### Top1 mRNA and protein expression levels show no difference between parental and SN-38 resistant cells

**Fig. 3:**
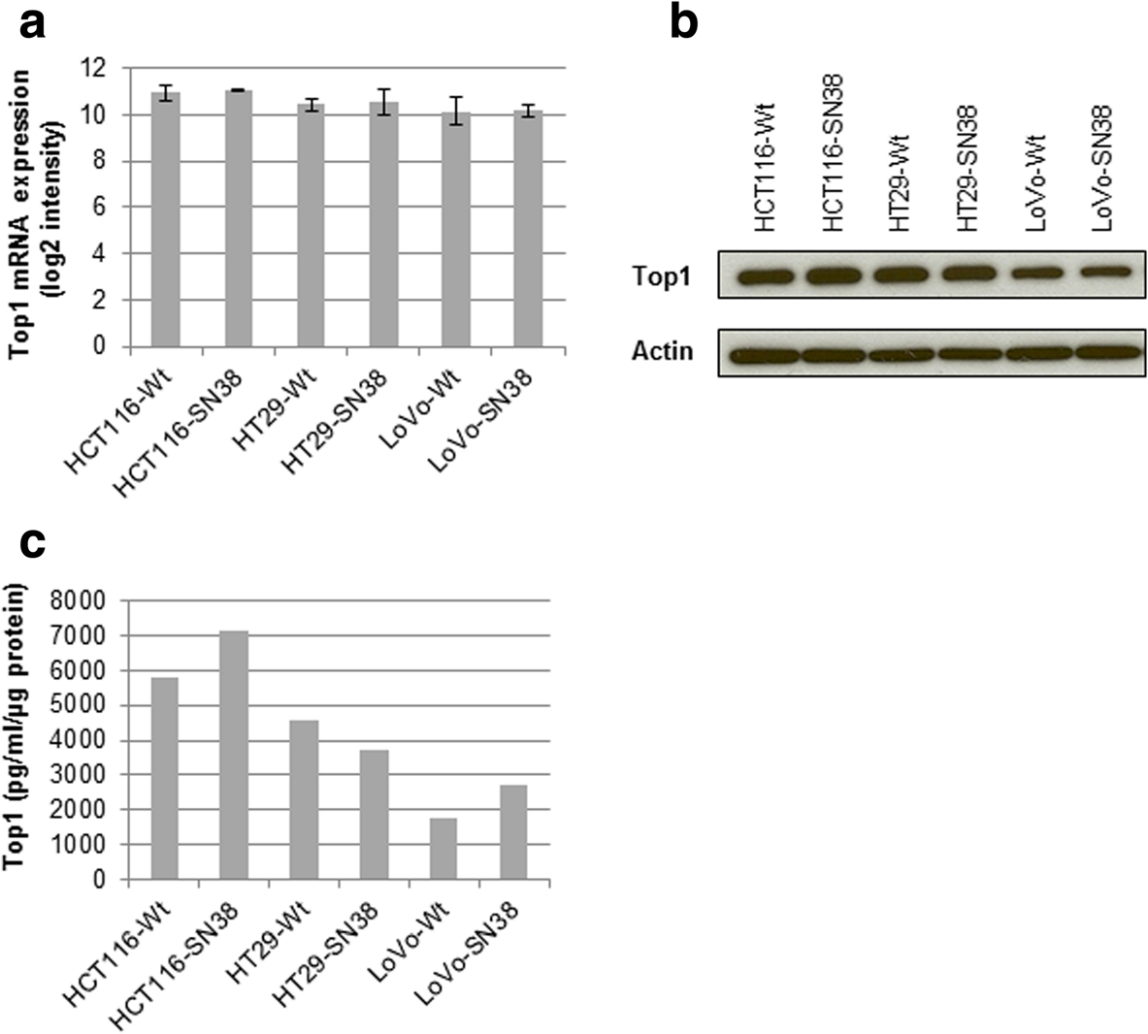
*TOP1* mRNA and protein expression in parental and SN-38 resistant cells. **a** mRNA expression levels (log2 transformed intensities), mean ± standard deviation. **b** Representative Western blot using anti-Top1 and anti-actin (loading control) antibodies (10 μg total protein were loaded per sample). **c** Top1 protein levels measured by ELISA assay

*TOP1* mRNA expression was determined by microarray analysis [36] (see Fig. 3a). No significant difference in mRNA level between corresponding parental and resistant cells was observed. In addition, Top1 protein expression levels, determined by Western blotting and ELISA (see Fig. 3b and c) were similar in the corresponding parental and SN-38 resistant cells.

### Top1 enzyme activity in the presence of drug is highly affected in SN-38 resistant cells

**Fig. 4:**
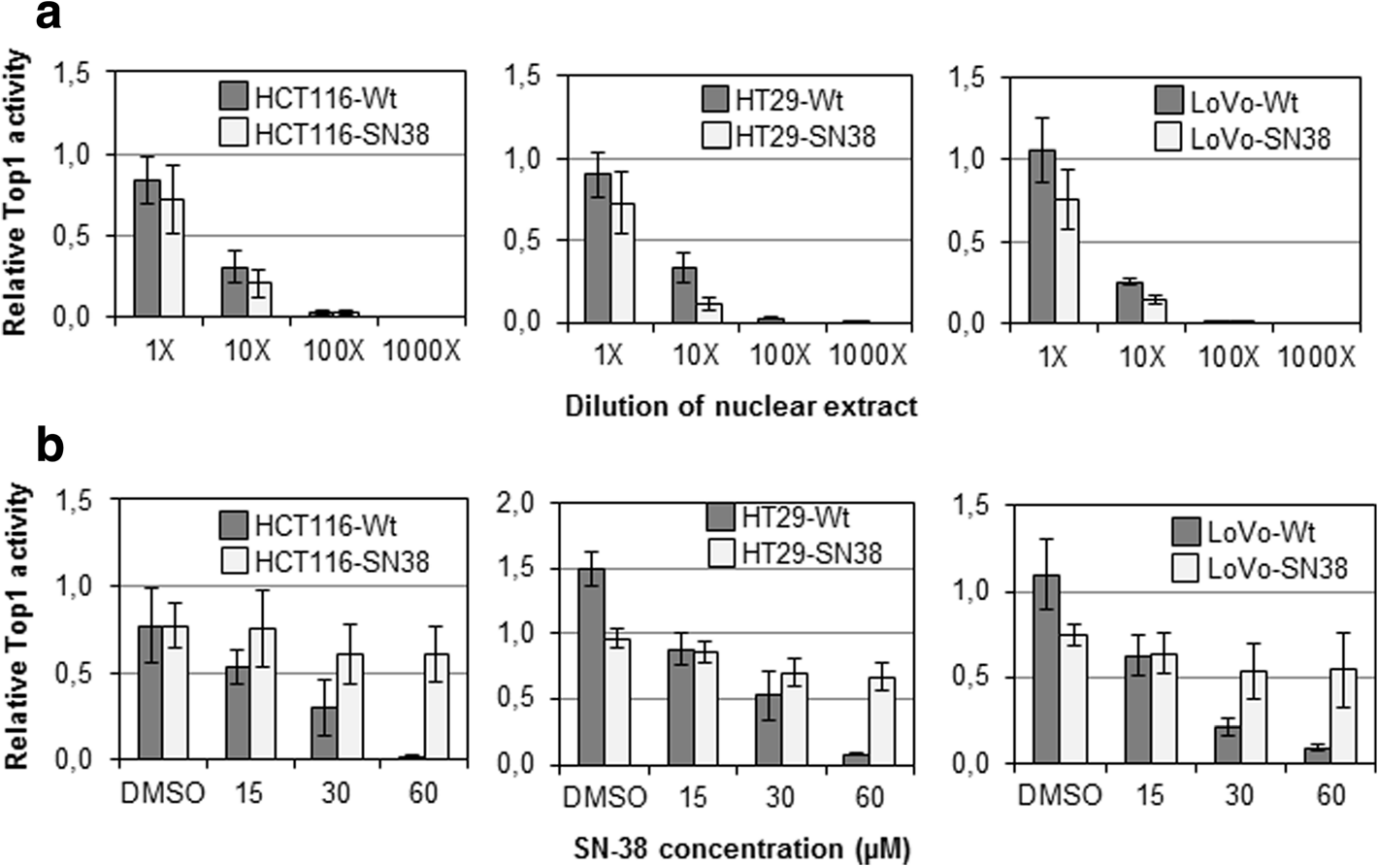
Enzymatic activity of Top1 in nuclear extract from parental and SN-38 resistant cells. **a** Top1 activity at different dilutions of nuclear extract in the absence of SN-38. **b** Top1 activity measured in the presence of increasing concentrations of SN-38. DMSO is the vehicle control without SN-38. Mean values ± standard deviations are plotted

The enzymatic activity of Top1 in nuclear extracts from parental and resistant cells was determined by the Rolling circle Enhanced Enzyme Activity (REEAD) assay previously described by Stougaard et al. [41] and Andersen et al. [40]. Top1 activity in the absence of drug determined at different dilutions of nuclear extract was found to be similar or slightly lower in the SN-38 resistant and corresponding parental cells (see Fig. 4a). Next, Top1 activity was measured in the presence of increasing concentrations of SN-38 (see Fig. 4b). In the parental cells, Top1 activity diminished with increasing concentrations of SN-38, while the Top1 activity in the SN-38 resistant cells was largely maintained with increasing SN-38 concentrations.

### Pattern of cross-resistance to non-camptothecin Top1-targeting drugs and Top2-targeting drugs

**Fig. 5:**
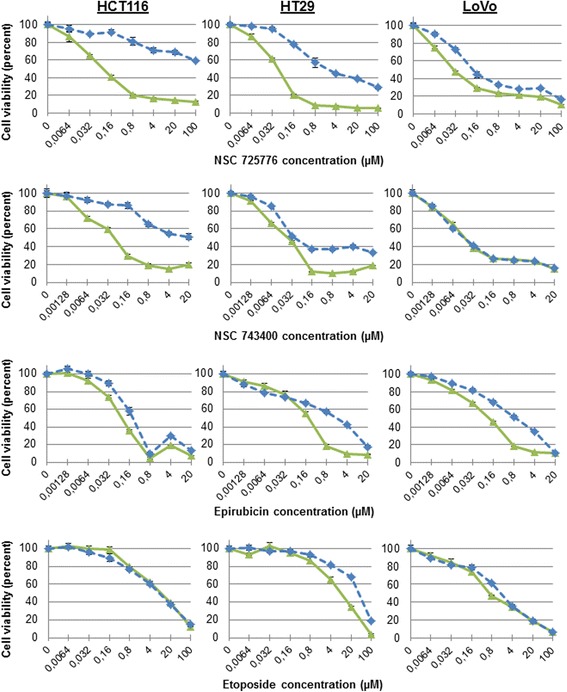
Drug sensitivity assays for parental and SN-38-resistant cells against two non-camptothecin Top1-targeting drugs (NSC 725776/LMP776 and NSC 743400/LMP400) in clinical trial and two clinical Top2-targeting drugs (epirubicin and etoposide). Green solid lines are the parental cells and blue dotted lines are the SN-38 resistant cells. Cells were exposed to drug for 48 h and cell viability determined using the MTT assay. Shown are representative results

The cross-resistance of the SN-38-resistant cells were determined against two indenoisoquinoline non-camptothecin Top1-targeting drugs in clinical trial; NSC 725776 (LMP776/indimitecan) and NSC 743400 (LMP400/indotecan), as well as two drugs targeting Top2; the anthracycline epirubicin and etoposide (see Fig. 5). IC50-values and relative resistances are shown in Table 3, including IC50-values for SN-38 for comparison. HCT116-SN38 displayed a strong resistance to NSC 725776 and NSC 743400, but was sensitive to epirubicin and etoposide. HT29-SN38 showed a moderate or strong resistance to NSC 725776 and epirubicin, respectively, but was relatively sensitive to NSC 743400 and etoposide. LoVo-SN38 displayed a moderate resistance to NSC 725776 and epirubicin, but was sensitive to NSC 743400 and etoposide.

**Table 3:** Drug sensitivity IC50-values and relative resistances

Cell line	SN-38		NSC 725776/LMP776		NSC 743400/LMP400		Epirubicin		Etoposide	
	IC50	RR	IC50	RR	IC50	RR	IC50	RR	IC50	RR
HCT116-Wt	0.05 ± 0.01	1	0.06 ± 0.03	1	0.17 ± 0.1	1	0.09 ± 0.01	1	6.3 ± 2.8	1
HCT116-SN38	3.4 ± 0.6	67	47 ± 46 ^a^	782	47 ± 46	280	0.2 ± 0.03	2.1	4.4 ± 3.5	0.7
HT29-Wt	0.13 ± 0.06	1	0.03 ± 0.01	1	0.07 ± 0.04	1	0.18 ± 0.02	1	9.9 ± 3.7	1
HT29-SN38	7.3 ± 1.7	55	1.2 ± 0.7	36	0.14 ± 0.04	2	2.0 ± 0.9	11	38 ± 17	4
LoVo-Wt	0.02 ± 0.004	1	0.02 ± 0.01	1	0.06 ± 0.02	1	0.11 ± 0.03	1	1.8 ± 1.9	1
LoVo-SN38	0.44 ± 0.2	20	0.09 ± 0.03	4.1	0.05 ± 0.03	0.8	0.95 ± 0.4	9	2.7 ± 1.6	1.5

Mean IC50-value (μM) ± standard deviation of three experiments. RR; relative resistance is the IC50-value of the resistant cell line divided by the IC50-value of the parental (wild-type, Wt) cell line

^a^Did not reach IC50, so the actual IC50-value is larger than this. IC50-values for SN-38 are provided for comparison

### Top1-DNA cleavage complexes formed after drug treatment is altered in SN-38 resistant cells

**Fig. 6:**
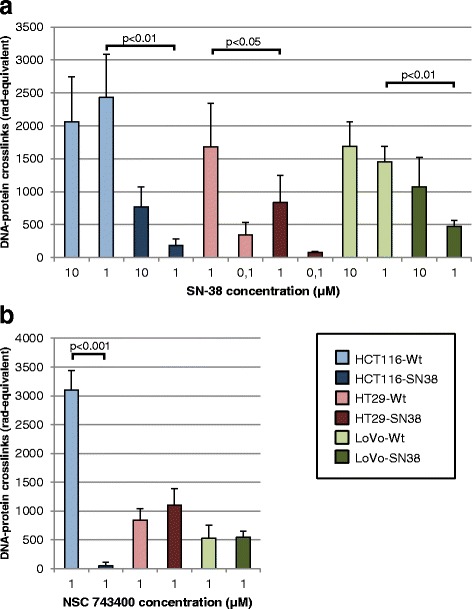
Detection of Top1-DNA cleavage complexes following drug treatment of parental or SN-38 resistant cells. Cells were exposed to drug for 1 h, either **a** SN-38 (10, 1 or 0.1 μM) or **b** NSC 743400/LMP400 (1 μM). Formed cleavage complexes were measured by alkaline elution and are given as DNA crosslinks (in rad-equivalent). Shown are mean values ± standard deviations of four replicates. *P*-values were calculated between corresponding parental and resistant cell lines treated with 1 μM of either SN-38 or NSC 743400

Parental and SN-38 resistant cells were treated with SN-38 or NSC 743400, and Top1-DNA cleavage complexes were assessed by alkaline elution (see Fig. 6). Firstly, cells were exposed for 1 h to SN-38. All three parental cells displayed a significantly higher number of Top1-DNA cleavage complexes than their corresponding SN-38 resistant cells, in line with the drug sensitivity data. Secondly, cells were exposed for 1 h to NSC 743400, as this drug showed strong cross-resistance in HCT116-SN38, but not in HT29-SN38 and LoVo-SN38. In line with this, significantly fewer cleavage complexes were formed in HCT116-SN38 cells compared to HCT116-Wt cells, whereas cleavage complexes were comparable in parental and resistant HT29 and LoVo cell lines.

## Discussion

As Top1 is the sole target of camptothecins [8, 43], Top1 alterations play a critical role in mediating resistance to irinotecan and as a possible predictive biomarker of response to irinotecan in mCRC. In the present study, we characterized the Top1 status in three human colon cancer cell lines with acquired resistance to the irinotecan’s metabolite SN-38, investigating Top1 gene copy number, genetic sequence, mRNA and protein expression, enzyme activity and formation of Top1-DNA cleavage complexes following drug treatment of cells. In addition, we investigated the cross-resistance to two non-camptothecin Top1-targeting drugs and two Top2-targeting drugs.

Compared to their sensitive parental counterparts, the SN-38 resistant cells did not show noticeable changes in the expression level of Top1, neither looking at mRNA or protein expression. There appeared to be a relatively good correlation between Top1 protein expression measured by Western blotting and ELISA. Furthermore, we found the Top1 activity in SN-38-resistant cells, in the absence of any drug treatment, to be either unchanged or slightly reduced compared to parental cells. In previous studies, camptothecin response has been associated with high Top1 protein expression [16, 24, 26] or high Top1 enzyme activity [15, 23–25]. Moreover, two studies found a high Top1 mRNA expression to be associated with camptothecin sensitivity [25, 44], while two others did not [15, 45].

Looking at the *TOP1* gene copy number in the SN-38 resistant cells using FISH, we identified changes in two of the three resistant cell lines (HCT116-SN38 and HT29-SN38). The first was a *TOP1* gene copy gain in a subpopulation of cells (about half of the cells) independently of centromere-20, a marker for the chromosome bearing *TOP1* (HCT116-SN38), while the other was a loss of one copy of chromosome 20 (including one copy of *TOP1*) (HT29-SN38). Two cell line studies by McLeod and Keith [21] and Romer et al. [17] demonstrated a positive correlation between *TOP1* gene copy number and sensitivity to SN-38 and between gene copy number and protein expression, respectively. The positive association between *TOP1* amplification and expression of Top1 mRNA and protein was confirmed by another study [22], and Top1 mRNA and protein expression have been shown to correlate well in the NCI-60 cancer cell line panel [14].

Using primers covering the full coding region of *TOP1* we did a mutational analysis of the gene in the SN-38 resistant and parental cells. Two of three resistant cell lines did not harbor any mutations, while the third (HCT116-SN38) harbored two mutations. Both identified mutations were non-synonymous and heterozygous; one located at amino acid residue position 364 (c.1336C > T, R364K, arginine to lysine change) in the Top1 core domain and the other at position 717 (c.2395G > A, G717R, glycine to arginine change) in the C-terminal domain [27, 46]. Both were shown by multiple sequence alignment to be located at highly evolutionarily conserved positions in the Top1 protein. Using *TOP1* wild-type and mutant specific primers, we showed by PCR that the mutated cells (HCT116-SN38) expressed mutant *TOP1* mRNA at a markedly higher level than wild-type *TOP1* mRNA, even though the sequencing analysis suggested heterozygosity of the mutations. *TOP1* containing a single mutation (R364K or G717R) appeared to be expressed at a higher level than double-mutant *TOP1*, suggesting that the mutations are present on separate alleles or in separate subpopulations in the HCT116-SN38 cells, each of them conferring resistance. This is in accordance with the FISH analysis, which detected two subpopulations in the HCT116-SN38 cell line. In line with these findings, we demonstrated that Top1 activity in nuclear extract from the HCT116-SN38 cell line was largely unaffected in the presence of high doses of SN-38, which completely eliminated the Top1 activity in parental cells, while the Top1 activity in drug absence was the same in HCT116-SN38 and parental cells. Furthermore, we measured the degree of formation of Top1-DNA cleavage complexes by alkaline elution following SN-38 treatment of cells, and showed that far fewer complexes were formed in HCT116-SN38 cells compared to parental cells. These findings suggest that SN-38 binding to Top1-DNA is hindered in the mutation-harboring cells. In previous studies, identified *TOP1* mutations have clustered in regions close to the structural site where Top1 binds DNA and camptothecin, i.e. the regions 361–364 (DNA minor groove), 503–533 (minor groove) and 717-729 (major groove) [11, 47]. Some mutations have been shown to hinder binding of drug, while others destabilize the drug-Top1-DNA cleavage complex or enhance Top1 DNA religation. Other mutations have also been identified in relationship to the linker region of Top1 [38, 48]. In a study by Li et al. [49] amino acids in the 361–364 region was demonstrated to be involved both in enzyme catalysis and camptothecin resistance. More specifically, experimental substitution of amino acids in the 361–364 range, i.e. R362L (arginine to leucine) and R364G (arginine to glycine) was shown to affect the catalytic activity of Top1, however R364G only slightly reduced the activity compared to wild-type enzyme [49]. Furthermore, Li et al. [49] showed that R364G Top1 was able to bind DNA with the same affinity as wild-type enzyme, however camptothecin was largely unable to bind R364G Top1-DNA cleavage complexes and cause DNA breaks. In addition, a R364H (arginine to histidine change) mutation was previously described in two camptothecin resistant prostate cancer cell lines [50]. The R364H mutation did not affect the catalytic activity, but rendered the cells resistant to camptothecin [50]. As lysine is a large positively charged amino acid, similar to histidine, it is very likely that R364K Top1 is functionally similar to R364H Top1. Several other mutations associated with camptothecin resistance have previously been reported in the amino acid region 361–365 of Top1 [51–55]. The C-terminal domain of Top1 is known to be involved in both enzyme catalytic activity and drug binding [27], and several camptothecin resistance-associated mutations have been reported in the 717–737 region [56–62], including a pair of mutations in a tumor sample from a cisplatin/irinotecan treated lung cancer patient [29]. A camptothecin-associated mutation at position 717 (G717V, glycine to valine change) has previously been reported together with the mutation T729I [56]. The authors showed that the mutation-harboring cells displayed similar Top1 catalytic activity as wild-type cells, and that each mutation on its own rendered yeast cells resistant to camptothecin [56]. Losasso et al. [62] investigated various amino acid substitutions in the 729 position, and suggested that this position is part of a hydrophobic pocket important for drug sensitivity [62]. Recently, mutations have also been reported in the linker region (amino acid residues 636–712), between the core and C-terminal domains of Top1 [38, 48]. Losasso et al. [48] investigated a resistance-associated mutation at position 653 and suggested that altered Top1 linker flexibility is a likely mechanism of resistance [48]. Gongora et al. [38] have subsequently identified other mutations in the linker region, which could confer resistance by this mechanism [38]. One of the linker region mutations found by Gongora et al. (Glu710Gly) was further analyzed in S. cerevisiae and the data indicated that a fully functional linker region of Top1 is important to confer camtotethecin sensitivity [63]. We did not detect any *TOP1* mutations in the two other SN-38 resistant cell lines (HT29-SN38 and LoVo-SN38). However, even in these cells Top1 activity was unaffected by the presence of large concentrations of SN-38, and thus other mechanisms must be responsible for this finding.

Lastly, we assessed the three SN-38 resistant cell lines for cross-resistance to two non-camptothecin Top1-targeting drugs in clinical trials (indenoisoquinolines: NSC 725776/LMP776/indimitecan and NSC 743400/LMP400/indotecan) and two clinical Top2-targeting drugs (epirubicin and etoposide). All three SN-38 resistant cell lines displayed no or very little cross-resistance to etoposide, a specific Top2 inhibitor [12, 13, 46], while showing more but still only partly cross-resistance to epirubicin, a DNA intercalating Top2 inhibitor [64]. Previous studies have demonstrated that reduced activity of Top1 can be compensated by increased activity of Top2 and thus increased sensitivity to etoposide [65, 66], and furthermore that camptothecin-resistant cells retain sensitivity to Top2-targeting drugs [50]. In the three SN-38 resistant cell lines, the indenoisoquinolines displayed an interesting pattern of resistance, from full cross-resistance to no cross-resistance. The two indenoisoquinolines are currently in clinical development [67]. In the present study, the cell line harboring the R364K-G717R mutations (HCT116-SN38) showed very strong cross-resistance to LMP400 and LMP776. The two SN-38 resistant cell lines, which did not carry any mutations in *TOP1* (HT29-SN38 and LoVo-SN38), were partly cross-resistant to LMP776, while showing either no or only small cross-resistance to LMP400. Studies in our laboratory [36] showed that HT29-SN38 and LoVo-SN38 both strongly upregulated expression (mRNA; 25- and 60-fold, respectively) of the well-known drug-efflux pump ABCG2 (BCRP) [68, 69], while HCT116-SN38 did not (data not shown). This suggests that LMP400 can remain active in cancers resistant to SN-38, which display upregulation of this multidrug resistance protein, if *TOP1* is wild-type. This is in line with a previous study by Antony et al. [35], where LMP776, but not LMP400, was shown to be a weak substrate of the ABCG2 pump. LMP400 was thus effective in cells overexpressing ABCG2, which displayed a 46-fold resistance to SN-38 [35]. Our experiment measuring Top1-DNA cleavage complexes following treatment with LMP400 supported the cross-resistance findings. These results highlight how the underlying molecular mechanism of camptothecin resistance in cancer cells determines their resistance-profile to new classes of drug such as the indenoisoquinolines.

## Conclusions

We generated three SN-38-resistant human colon cancer cell lines and investigated Top1. We detected no difference in the expression level of Top1 and no to very little reduction in Top1 activity in the absence of drug. A markedly increased activity of Top1 in the presence of SN-38 was seen in all three resistant cell lines. *TOP1* gene aberrations were detected in two of three cell lines, and a not previously reported pair of mutations in *TOP1* was identified in one cell line. The SN-38 resistant cells displayed an interesting pattern of cross-resistance to two indenoisoquinoline Top1-targeting drugs: SN-38 resistant cells with mutant *TOP1* and no overexpression of drug-efflux pump ABCG2 were resistant to LMP400, while SN-38 resistant cells with wild-type *TOP1* and overexpression of ABCG2 remained sensitive to LMP400. Furthermore, cross-resistance to Top2-targeting drugs was not existent or limited. Thus, this study adds to the growing knowledge about anti-cancer resistance mechanisms for camptothecins and the new class of indenoisoquinoline Top1-targeting drugs.

## Abbreviations

CEN-20: centromere-20
mCRC: metastatic colorectal cancer
mt: mutant
Top1: DNA topoisomerase I
Top2: DNA topoisomerase II
wt: wild-type
